# Nitrate Poisoning due to Ingestion of Cabbages (*Brassica oleracea* var. *capitata* L.) (Brassicaceae) in Kitui County, Kenya

**DOI:** 10.1155/2019/8716518

**Published:** 2019-10-09

**Authors:** Joseph Mwanzia Nguta

**Affiliations:** Department of Public Health, Pharmacology and Toxicology, Faculty of Veterinary Medicine, University of Nairobi, Nairobi, Kenya

## Abstract

Nitrate toxicosis associated with consumption of cabbages (*Brassica oleracea var. capitata* L.) (Brassicaceae) was diagnosed in a small herd comprising of ten goats in Matinyani ward, Kitui West sub-County of Kitui County in Kenya. The clinical signs were ataxia, ruminal tympany, bluish-brown mucous membranes, rapid and difficult breathing, incoordination, head pressing, aggressive movements, jugular distension, increased heart rates, tremors, and coma. Six goats died of intoxication. Dark brown/chocolate-colored and poorly clotted blood was the prominent necropsy finding. There was limited froth in the trachea and bronchi consistent with mild pulmonary oedema. There were liver congestion, pericarditis, and enlarged and congested kidneys with pinpoint haemorrhages. Four other affected goats were treated with intramuscular antihistamines and oral administration of ivermectin. No further mortality was observed after the treatment. Cabbages fed to goats had 6.6% nitrate on dry matter (DM) basis , while ruminal contents had 5.55% nitrate on dry matter basis (DM). Dark brown blood collected at postmortem had a methemoglobin fraction of 78%. Forage nitrate levels of 0.5% and above are potentially dangerous, with acute poisoning likely to occur if the nitrate level exceeds 1%. Death can occur when blood methemoglobin levels rise up to 67–90%. Findings from the current study are indicative of nitrate-nitrite toxidrome associated with ingestion of cabbages.

## 1. Introduction

Livestock poisoning from nitrate exposure can be a major cause of morbidities and mortalities in farming systems [1, 2]. The median lethal dose (LD_50_) of nitrate in cattle appears to be approximately 0.5 g/kg of body weight when fed as a part of the animal feed. However, the LD_50_ of nitrate was found to be much lower following oral administration as a drench, suggesting that the route of exposure and nitrate formulation play a key role in mediation of nitrate toxicity [3]. Acute nitrate intoxication has also been shown to be a function of dietary composition since livestock diets rich in carbohydrates have been associated with decreased nitrate poisoning [4]. Forage nitrate levels of 0.5% and above are potentially dangerous, with acute poisoning likely to occur if the nitrate level exceeds 1% [5]. Scientists have considered pastures with nitrate in the range of 0.34% to 0.45% nitrate-nitrogen (1.5–2.0% nitrate) as potentially toxic [6], while other investigators [7] reported that cattle fed with a poor or a diet deficient in carbohydrates died from a nitrate level of 0.7% [7]. Nitrate levels of 1% in forages on dry matter (DM) basis may be expected to result in acute toxicity and death in livestock [8]. When consumed more rapidly than they can be converted in the rumen to protein, nitrates enter the bloodstream as nitrite, which combines with hemoglobin in red blood cells to produce methemoglobin, a form incapable of transporting oxygen. Death occurs as a result of asphyxiation as methemoglobin levels approach 80% [9].

Ruminants exhibit the highest vulnerability to nitrate poisoning due to the nitrate reducing potential of rumen microorganisms to potentially toxic nitrites. Nitrates (NO_3_^–^) and nitrites (NO_2_^–^) are naturally occurring ions; both are products of oxidation of nitrogen (N) by microorganisms in plants, soil, or water and, to a lesser extent, by lightening. Nitrate is the more stable form of oxidized N but can be reduced by microbial action to nitrite [10]. The reduction of nitrate (NO_3_^–^) to more toxic nitrite (NO_2_^–^) is an intermediate step in the bacterial biochemical sequence of the formation of fully reduced ammonia (NH_3_) which is used to form microbial proteins. Toxicosis by ammonia does not occur in ruminants exposed to nitrate-containing feeds [11, 12] since the amount of ammonia produced is limited. In case of rapid ingestion of forages containing high nitrate levels, nitrite will accumulate in the rumen. Nitrite ions are subsequently absorbed through the rumen epithelium into the blood, where they passively enter into the erythrocytes in exchange for chloride ions. The nitrite ion in the red blood cells oxidizes hemoglobin to form methemoglobin which is incapable of transporting oxygen to body tissues, resulting in hypoxia. Signs of anoxia are usually observed when 30–40% of hemoglobin is converted to methemoglobin, with mortalities occurring when methemoglobinaemia is characterized by more than 80% of methemoglobin [13, 14]. Abortion in ruminants due to the fetal hypoxia is a sequel of nitrate intoxication and usually occurs three to seven days after exposure of pregnant dams to toxic nitrate levels [13, 14].

The most common source of nitrate toxidrome comprises plants containing accumulated toxic nitrate levels. A number of plants contain levels of nitrates that may be converted in the rumen to nitrites sufficient to cause intoxication. These plants include cabbages, turnips, sugar beets, lettuce, potatoes, and carrots which tend to concentrate nitrate ions, especially if they are grown using a high application of N fertilizers or grown in well-fertilized soils with manure or if grown during drought. In addition to nitrate toxicosis, induction of goiter has been associated with consumption of large quantities of uncooked cabbages [15]. Crops such as corn (*Zea mays* L. (Poaceae)), small grains, especially pearl millet (*Pennisetum glaucum* (L.) R. Br. (Poaceae)), sudan grass (*Sorghum bicolor subsp. drummondii* (Nees) de Wet (Poaceae)), and sorghum (*Sorghum bicolor* (L.) Moench (Poaceae)) can accumulate toxic nitrate levels [5]. Weeds such as redroot pigweed (*Amaranthus retroflexus* L. (Amaranthaceae)), lambsquarters (*Chenopodium album* L. (Amaranthaceae)), jimson weed (*Datura stramonium* L. (Solanaceae)), variegated thistle (*Silybum marianum* (L.) Gaertn. (Asteraceae)), sunflower (*Helianthus annuus* L. (Asteraceae)), and bindweed (*Convolvulus arvensis* L. (Convolvulaceae)) can also accumulate large quantities of nitrates under adverse weather conditions [5, 16]. The greatest potential for toxic levels of nitrates exists in unripe plants and plant parts that are regrown after harvest, such as green potatoes. Factors related to plants and animals have important roles in the development of toxicosis and its outcome [13, 17]. For instance, herbicide application, especially phenoxy derivatives of fatty acids, can enhance nitrate accumulation in plants. The concentration of nitrates in cabbages can vary considerably and may be as much as 1.2 g/kg fresh weight and these levels have the potential to cause toxic effects in ruminants [18]. Nitrites induce poisoning due to fatal anoxia when methemoglobin, with a much lower capacity to carry oxygen, is formed [19]. A suspected case of nitrate poisoning in a herd of ten goats fed on cabbages is reported. The case was reported by the client to the local veterinary personnel approximately thirty minutes after feeding goats with approximately twenty five kilograms (25 kgs) of cabbage leaves collected from Kalundu, a nearby market in Kitui West sub-County of Kitui County in Kenya.

## 2. Materials and Methods

The presented patients were adult Maasai goats comprising eight females and two males and are localized in Kitui central sub-County, Kitui County. The goats were reported to browse on their own in the field. The animal attendant indicated that all the goats were regularly dewormed with 10% albendazole and were treated monthly against external parasites with Bayticol. The goats had no history of disease and were in good body condition prior to exposure to the cabbages which they had not been previously fed on. In mid-April 2019, a small herd of ten adult Maasai goats in Matinyani ward, Kitui west sub-County, Kitui County, were fed with cabbages that had been collected from Kalundu, a nearby shopping centre in Matinyani ward, Kitui west sub-County, Kitui County in Kenya. Urea and potassium nitrate soil fertilizers are reported to be regularly used to grow cabbages in the fields in Kalundu area. About two hours after cabbage consumption, all the ten goats showed clinical signs of poisoning including ruminal tympany, foam in the mouth, weakness, unsteady gait and incoordination, head pressing, aggressive movements, and biting on objects. Six goats (60%) which exhibited dyspnea, tremors, ataxia, rapid heartbeat, and terminal convulsions died following a short course of disease. Four goats (40%) which presented mild toxicosis showed jugular distention, ruminal tympany, hypermetria, incoordination, tachycardia, dyspnea, and sunken eyes. Their mucous membranes were pale to purple.

Presumptive diagnosis of nitrate poisoning was made on the basis of acute nature, clinical signs, and history of cabbage ingestion. Dark brown/chocolate-colored venous blood samples were obtained from the affected goats. The diphenylamine blue test confirmed the presence of nitrates in cabbages, whole blood, ruminal contents, the liver, and the kidney. Urine samples were positive for nitrites when tested by a urine test strip (Combi-Screen, Germany). CO-oximetry performed on a radiometer 625 ABL showed a methemoglobin (MetHb) fraction of 78%. Two samples of cabbage leaves were taken to the Department of Public Health, Pharmacology and Toxicology (PHPT), a toxicology laboratory for analysis of nitrate content. Methylene blue, the antidote of nitrate poisoning, was not available then, and four affected goats were treated with intramuscular injection of antihistamines as well as oral administration of ivermectin. Fifty milliliters of 10% oxy-tetracycline was also administered intraruminally. There was no further mortality after treatment. Analysis of cabbage and ruminal content samples by radiopotentiometry revealed 6.6% and 5.55% nitrate in dry matter (DM) basis, respectively, confirming the diagnosis of nitrate poisoning in the affected goats. One day after poisoning, all the four treated goats had completely recovered.

## 3. Discussion

Nitrate intoxication has been reported to mainly occur in cattle found in Western countries [20]. However, in spite of abundance of nitrate accumulator plants [21], data on nitrate poisoning in Kenyan livestock are scarce. Nitrate is found in majority of plants in varying concentrations, but can accumulate to toxic levels under certain conditions, such as drought, cloudy weather conditions, and at night [20]. Nitrate can also accumulate in forages following excessive application of nitrogen-containing fertilizers.

In the present report, repeated use of nitrogen fertilizers and drought could have resulted in high levels of nitrate in cabbages which were fed to the goats. Nitrate levels higher than 15,000 ppm in dry matter (DM) in forages are toxic to goats, sheep, and cattle [13, 17]. The forage nitrate levels of 0.5% and above are potentially dangerous, with acute poisoning likely to occur if the nitrate level exceeds 1% [5]. Outbreaks of nitrate poisoning are usually recorded following exposure of goats to forage containing 3–7% in DM of potassium nitrate [12]. This observation is in agreement with the findings from the current report, where six goats died following exposure to cabbages containing 6.6% of nitrate on dry matter basis. Nitrate accumulates in the vegetative tissue, particularly in stalks and low amount in the leaves. Seeds do not generally contain toxic nitrate levels. Heavy fertilization of pastures, herbicide treatment, drought, cloudy weather, and decreased temperature may increase the nitrate concentrations in plants [5]. This observation is in part supported by findings from the current report, where nitrate-containing fertilizers, herbicides, and pesticides had been used. Nitrate toxicity in ruminants is influenced by a number of factors including species, the amount of carbohydrate in the diet, adaptation to nitrate in the diet, and the rate of nitrate consumption. In the current case report, the intoxicated goats did not have prior exposure to cabbages rich in nitrates. In addition, the goats were exposed to large quantities of cabbages containing toxic nitrate levels that could have played a central role in the observed poisoning. The most important factor underlying nitrate toxicity appears to be the rate of ingestion of the nitrate-containing forages. In the current report, the poisoned goats had not eaten any other forage prior to exposure to cabbages containing high nitrate concentrations. Cattle on high energy ration can also tolerate more nitrates [13, 17], and this could be the plausible explanation for the increased susceptibility of goats in the current report since the intoxicated goats had no access to an energy-rich ration.

Cabbages are among the different species of nitrate-accumulating plants. It is a common vegetable readily grown along the river banks by all Kenyan communities. Cabbage is an important vegetable known to mankind for over 4,000 years. It is a member of the mustard or cruciferous family (*Brassicaceae*), which includes mustard, rape, turnip, wasabi (*Eutrema wasabi*), radish, watercress, many oriental vegetables, and a very important model plant *Arabidopsis thaliana*.

Healthy animals have normal methemoglobin levels that are relatively constant at 2–3% of total hemoglobin [22]. When high nitrate feeds are consumed, moderate nitrate poisoning symptoms appear and 20–40% of the hemoglobin is converted to methemoglobin [23, 24]. Clinical signs reported in the current study are consistent with nitrate-nitrite poisoning. These signs reflect tissue oxygen deprivation and have been reported to become apparent when 30–40% of hemoglobin is converted to methemoglobin, which is incapable of oxygen transport [13, 14], with death occurring as a result of asphyxiation as methemoglobin levels rise up to 67–90 percent [9]. This observation is in agreement with results from the current report where methemoglobin (MetHb) fraction of 78% was recorded in dark brown venous blood collected from the goats at postmortem. Nitrate poisoning is primarily a problem in ruminants because of the reduction of nitrate to nitrite by ruminal microorganisms. The nitrite ion produces methemoglobin, which cannot react with oxygen, so anoxia occurs [8]. Methemoglobin leads to dark brown or chocolate-colored blood, a common feature of nitrate/nitrite poisoning. Clinical signs of acute nitrate poisoning include diarrhea, salivation, dyspnea, tremors, ataxia, rapid heartbeat, and terminal convulsions. Death may occur within 6–24 hours [8]. These observations are in agreement with current findings where affected goats exhibited difficulties in breathing and had dark brown blood with death being reported within six to twenty-four hours after exposure.

Clinical signs such as head pressing, aggressive behavior, and hypermetria which develop consequent to the central nervous system (CNS) dysfunction and reported in the present clinical case may also be related to severe CNS deprivation of oxygen and resultant hypoxia. Diagnosis of nitrate intoxication is based on observed clinical signs, chocolate/dark brown blood, and exposure to the toxic plant. Diagnosis of nitrate toxicosis can also be confirmed by analysis of the suspected forage or body fluids including urine and serum in live animals and the cerebrospinal fluid or aqueous humour in dead animals [13, 14]. Cabbages fed to goats had 6.6% nitrate on dry matter (DM) basis , while ruminal contents had 5.55% nitrate on dry matter (DM) basis . Dark brown blood collected at postmortem had a methemoglobin fraction of 78%. The forage nitrate levels of 0.5% and above are potentially dangerous, with acute poisoning likely to occur if the nitrate level exceeds 1%. Death can occur when blood methemoglobin levels rise up to 67–90 percent [5]. These findings are in agreement with observations from the current report where 60% of the affected goats died following consumption of cabbages with 6.6% nitrate. Methylene blue is the antidote of choice in ruminants with suspected nitrate poisoning. Ascorbic acid is an alternative antidote, but it has a slow onset of action. Intravenous administration of anticholinergic agents such as antihistamines reverses cholinergic effects associated with nitrate intoxication, while intravenous injection of vasoconstrictor agents such as epinephrine is helpful in reversing hypotension, even in cases that are at the point of death [13]. Intraruminal administration of antibiotics also suppresses the reduction of nitrate to nitrite.

As a conclusion, in case of acute mortality in goats fed with cabbages, nitrate poisoning should be considered, especially if the cabbages are grown under adverse weather conditions in well-manured farms or when nitrogen fertilizers have been applied. To support any suspicion and to facilitate follow-up, nitrate levels in the forage should be analyzed. In addition, the diphenylamine blue test and nitrate dipstick tests are highly recommended. If possible, serum from surviving cases should be considered. After methylene blue and antibiotic administration immediately following observation of clinical signs, majority of affected goats will recover. However, goat owners should take precaution while feeding their goats on cabbages to minimize cases of nitrate poisoning.

## Data Availability

The qualitative and quantitative data used to support the findings of this study are included within the article.

## Supplementary Materials

Supplementary Materials The images show gross pathology following postmortem examination.
