# Supplementary material for: Nitrate Poisoning due to Ingestion of Cabbages (Brassica oleracea var. capitata L.) (Brassicaceae) in Kitui County, Kenya
Source: ScientificWorldJournal. 2019 Oct 9;2019:8716518. doi: 10.1155/2019/8716518 (PMC6803730; doi:10.1155/2019/8716518)
Supplement: Supplementary Materials — The images show gross pathology following postmortem examination. [file 8716518.f1.pdf]

Supplementary materials

Figure one shows a post-mortem illustration of the digestive tract of one of the goats that died following ingestion of cabbages purchased from Kalundu market. Cabbages that were fed to the goats can be seen in the feeding area (**A**). Ruminal contents (**B**), dark brown/chocolate colored blood (**C**), pin point haemorrhages (**D**) are illustrated in the post-mortem image.

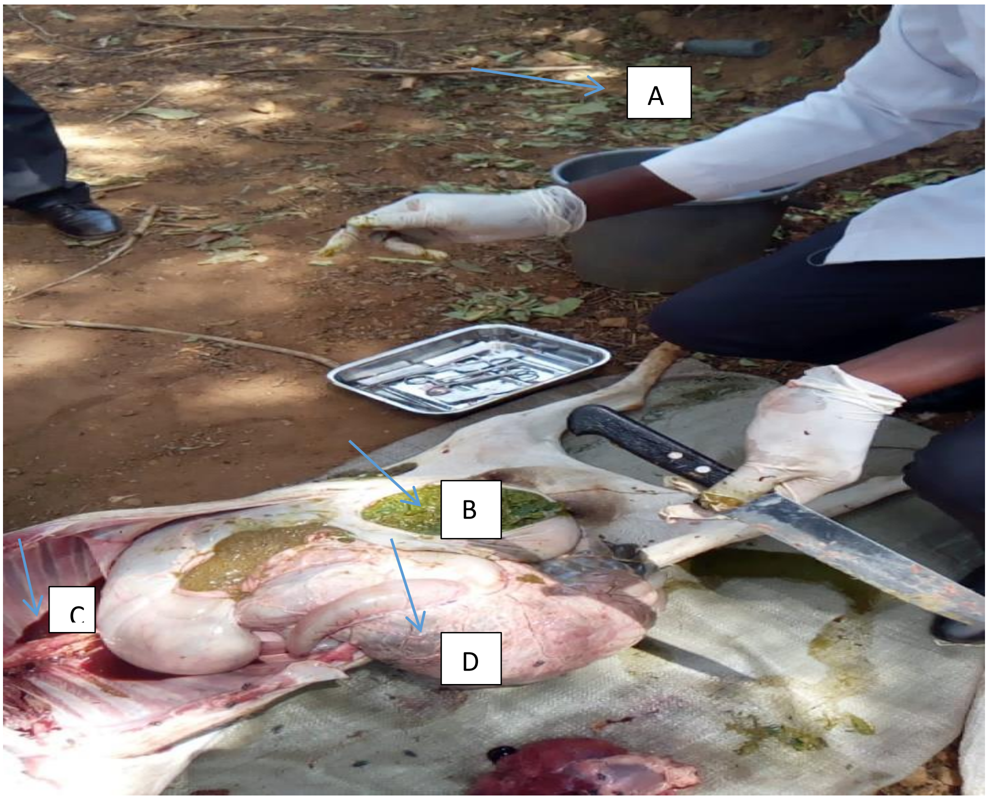

Figure 1: A post-mortem image of an an adult male Maasai goat showing ruminal contents, dark brown/chocolate colored blood and cabbages in the feeding area.

Figure two is a post-mortem illustration of the respiratory tract of a goat that died of suspected nitrate-nitrite poisoning. There was froth in the trachea (**A**), bronchi (**C**) and also in bronchioles (**C**). Dark brown/chocolate colored blood (**B**) could be observed.

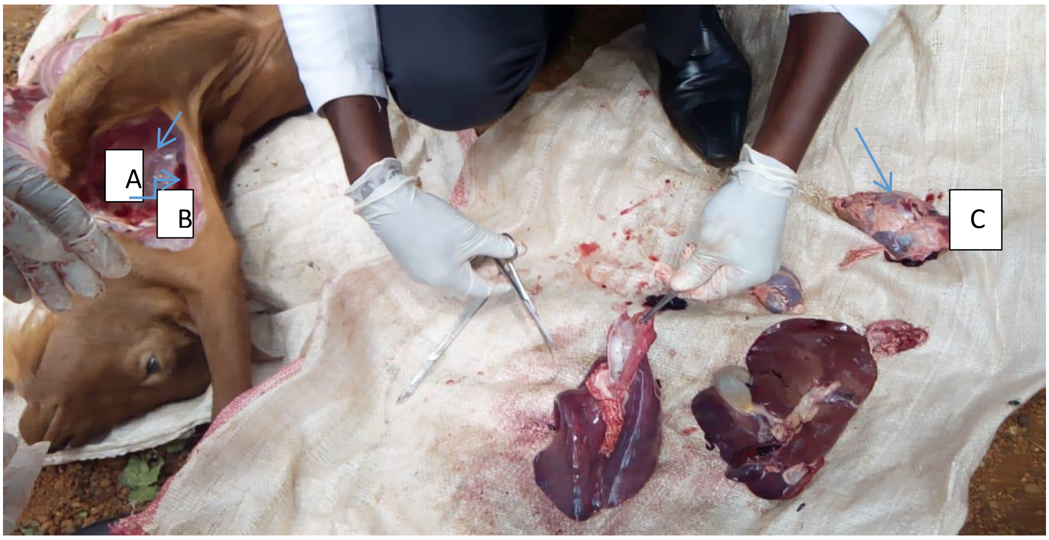

Figure 2: Post-mortem image of the respiratory tract of a goat that died of suspected nitrate-nitrite poisoning with froth in the trachea, bronchi and bronchioles. The dark brown blood is poorly clotted.

Figure three shows a post-mortem illustration of the kidneys from a goat that died from suspected nitrate-nitrite poisoning following ingestion of cabbages with 6.6% nitrate. The kidneys are slightly enlarged and congested (**A**) with pin point haemorrhages (**B**). The chocolate colored blood is poorly clotted (**C**).

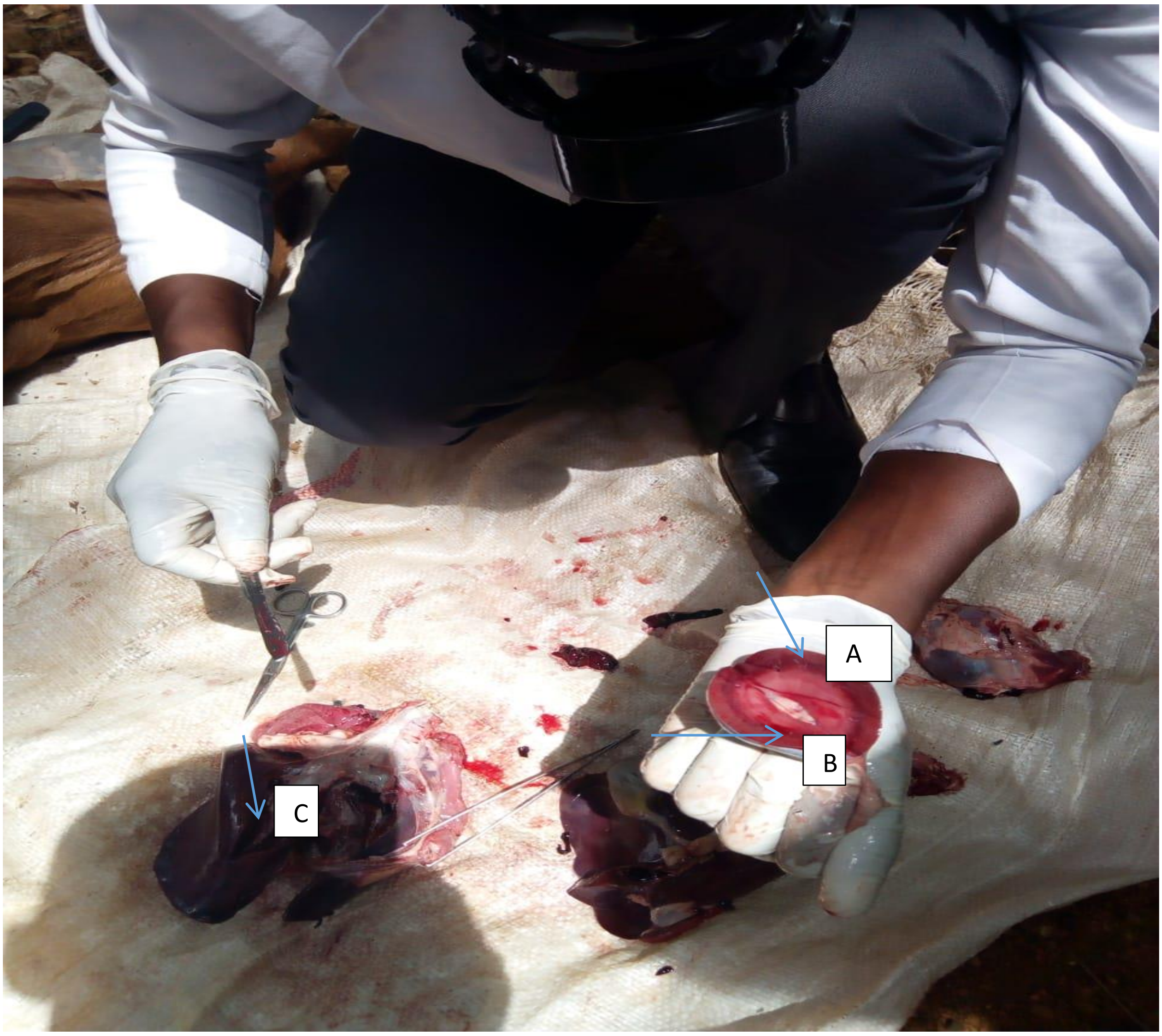

Figure 3: Postmortem image of the kidneys from one of the goats that died of suspected nitrate-nitrite poisoning following consumption of cabbages with 6.6% nitrate. The kidneys are slightly enlarged, congested with pin point haemorrhages.
